# Hair Number per Follicular Unit as a Marker of Treatment Response to Combined Autologous Scalp‐Derived Micrografts and Allogeneic SHED‐CM in Male Androgenetic Alopecia

**DOI:** 10.1111/jocd.70982

**Published:** 2026-06-17

**Authors:** Tomoko Kamishima, Ai Minamimura, Chie Hirabe, Junichi Taguchi

**Affiliations:** ^1^ Department of Dermatology Tokyo Midtown Skin Aesthetic Clinic Noage Tokyo Japan; ^2^ Tokyo Midtown Clinic Tokyo Japan

## Abstract

**Background:**

Autologous scalp‐derived micrografts (MG) and allogeneic dental pulp stem cell‐derived conditioned medium obtained from stem cells from human exfoliated deciduous teeth (SHED‐CM) have individually shown efficacy in treating male androgenetic alopecia (MAGA).

**Objectives:**

To evaluate the efficacy and safety of combined MG and SHED‐CM treatment (MGCM) and to identify trichoscopic parameters associated with inter‐individual variability in treatment responses.

**Methods:**

Fifty‐nine patients with vertex type MAGA received one MG session and four SHED‐CM injections. Global and hair‐whorl images, along with quantitative trichoscopic parameters, were assessed at baseline and 12 months. Overall response was evaluated using a composite Quantitative Trichoscopic Evaluation System (QTES) Score derived from representative trichoscopic parameters.

**Results:**

MGCM achieved a response rate of 69.5% without serious adverse events. Mild, self‐limited injection‐site pain and petechiae were noted in 33.9% of patients. Responders showed increases in hair diameter and hair number per follicular unit (HN/FU), whereas non‐responders exhibited a decline in HN/FU. HN/FU most clearly discriminated responders from non‐responders, both individually and within the composite QTES Score. Inter‐assessor reliability demonstrated good to excellent agreement.

**Conclusions:**

MGCM was well‐tolerated and resulted in clinically meaningful improvement in a substantial proportion of patients with MAGA. Changes in HN/FU, integrated within the composite QTES Score, provide a practical and appearance‐relevant marker of treatment response. These findings support the use of integrated quantitative trichoscopic assessment for monitoring and predicting treatment outcomes.

## 1 Introduction

Autologous scalp‐derived micrografts (MG) [1, 2, 3, 4] and allogeneic conditioned medium derived from stem cells from human exfoliated deciduous teeth (SHED; SHED‐CM) [5, 6] have each demonstrated therapeutic potential in the treatment of male androgenetic alopecia (MAGA). These two modalities represent fundamentally distinct regenerative strategies, differing primarily in their reliance on cell‐based versus cell‐free mechanisms.

In the MG approach, autologous scalp tissue fragments are mechanically disaggregated to generate a cell suspension containing viable follicular progenitor cells and hair follicle‐associated mesenchymal cell populations [1, 2, 3]. This suspension is subsequently injected into areas of scalp thinning. MG has been proposed to support tissue regeneration by enhancing cell proliferation and migration, facilitating intercellular signaling, and promoting remodeling of the extracellular microenvironment [7, 8, 9]. Mechanistically, MG is thought to stimulate the hair follicle niche, including activation of Wnt/β‐catenin signaling pathways involved in anagen induction [7] and the promotion of cell proliferation via ERK‐dependent signaling pathways [8]. Clinically, patients treated with MG have shown signs of follicular regeneration and improved hair growth [1, 2, 3, 4]. In a placebo‐controlled study, an increase of 23.3 hairs/cm^2^ over 12 months was observed, accompanied by both patient‐ and physician‐assessed global improvements in MAGA [3].

In contrast, conditioned medium (CM) represents a completely cell‐free therapeutic approach that supplies a diverse array of growth factors (VEGF, HGF, IGF‐1) and exosomes that synergistically promote angiogenesis and suppress micro‐inflammation [5, 6, 7, 10, 11, 12]. Evidence from recent studies suggests that CM and related cell‐free extracts promote hair regeneration through several complementary mechanisms, including activation of Wnt/β‐catenin signaling, enhancement of dermal papilla cell proliferation and survival via ERK and AKT pathways, and stimulation of perifollicular angiogenesis, alongside anti‐inflammatory effects. These coordinated actions are thought to facilitate anagen induction and improve follicular function in androgenetic alopecia [10, 11, 12].

Among various mesenchymal stem cell‐derived CM, SHED‐CM has attracted particular attention. Prior studies have shown that SHED‐CM induces hair growth more rapidly and effectively than other mesenchymal stem cell‐derived conditioned medium, notably by accelerating the transition from telogen to anagen in vivo [6]. On the basis of this comparative advantage, SHED‐CM was selected as the CM component in the present study.

Thus, MG may be characterized as a stem cell‐oriented, cell‐based regenerative therapy, delivering cellular “raw material” into the scalp to potentially rebuild hair follicle structures. In contrast, SHED‐CM represents a cell‐free, paracrine‐based therapy, acting primarily by modulating the follicular microenvironment and stimulating endogenous cellular responses. Each approach has distinct advantages and limitations. MG may be limited by cell viability, engraftment efficiency, and procedural invasiveness [7]. SHED‐CM, by avoiding cell transplantation, may offer a safer and simpler therapeutic option, although its efficacy may depend more strongly on the responsiveness of the recipient's naïve cells and vascular supply [7, 10]. Therefore, we hypothesized that MGCM combination therapy may exert synergistic effects by integrating the structural remodeling and Wnt‐pathway activation associated with MG with the potent angiogenic and paracrine effects of SHED‐CM.

However, despite the promise of such combination therapy, it remains unclear which quantitative trichoscopic parameters—such as hair density, hair shaft diameter, and follicular unit composition—are most sensitive to treatment‐induced changes or which patient characteristics best capture therapeutic responsiveness. Therefore, we evaluated the efficacy and safety of combined MG and SHED‐CM in patients with MAGA and aimed to identify quantitative trichoscopic parameters that best reflect treatment response and help distinguish responders from non‐responders.

## 2 Methods

### 2.1 Study Design

This was a single‐center, prospective observational cohort study conducted between October 2021 and March 2025. The study focused on male patients with vertex‐predominant hair thinning characteristic of MAGA.

### 2.2 Patient Selection and Ethics

Male patients diagnosed with MAGA were enrolled. Following dermatological examinations, physical assessments, and blood testing to exclude serious systemic diseases or other forms of alopecia, we defined two groups: an untreated group (*N* = 133) and a treatment group receiving combined MG and SHED‐CM (*N* = 59).

Within the treatment group, patients were categorized according to prior treatment history. Those classified as treatment‐free were defined as having either no prior treatment or only minimal exposure of less than one month, regardless of treatment type. Because clinically meaningful pharmacologic effects generally require several months of continuous treatment, substantial effects from short‐term exposure of less than one month were considered less likely, although potential residual influences on baseline hair characteristics cannot be excluded [13, 14].

Patients in the MGCM group were either entirely treatment‐free, conventional therapies for less than one month, or had undergone prior treatment without achieving improvement for at least one year in their MAGA symptoms before starting MGCM treatment.

To minimize the influence of previous therapies, the treatment‐free status of the cohort was strictly documented. Of the 59 patients, 37 (62.7%) were treatment‐free and had never received any therapy for MAGA. Among the remaining patients, 14 had used conventional therapies (minoxidil or finasteride) for less than one month, and 8 had used them for over one year without clinical improvement (defined as decreased QTES score prior to enrollment). All prior treatments were discontinued upon enrollment.

This group received one session of autologous MG and four sessions of SHED‐CM injections, and their pre‐ and post‐treatment conditions were evaluated.

All patients provided written informed consent for participation and publication of clinical images. The study protocol was approved by the Center Ethics Review Board, registered with the University Hospital Medical Information Network Clinical Trial Registry, and conducted in accordance with the Declaration of Helsinki.

### 2.3 Visual Assessment

To assess the progression of MAGA in visual appearance, we used Hamilton‐Norwood classification (H‐N C) based on global scalp images [15, 16] at baseline and 12 months after treatment. Evaluations were independently conducted by two dermatologists.

### 2.4 Trichoscopic Measurement

To examine the hair details, a quantitative trichoscopic evaluation was performed at baseline and 12 months. All enrolled patients (*N* = 59) were classified as having vertex‐thinning type MAGA.

Trichoscopic measurements were obtained within a 5 × 5 mm area centered on the vertex hair‐whorl, an anatomically stable region commonly affected in early MAGA [17]. As a developmentally determined structure with a fixed spatial location throughout life, the hair‐whorl provides a reliable anatomical landmark for longitudinal assessment. The 5 × 5 mm sampling area was selected to allow accurate visualization and reliable counting of individual hair shafts while maintaining sufficient follicular representation for quantitative trichoscopic analysis.

To further enhance positional reproducibility, images were obtained using standardized vertex positioning and consistent camera orientation across all visits, with the vertex hair‐whorl serving as a reference landmark. However, as measurements were confined to this predefined vertex region, the findings should be interpreted as reflecting localized changes and may not fully capture global scalp involvement.

Trichoscopic images were acquired using a dermocamera (DZ‐D100, CASIO, Japan). Then, detailed hair analysis was conducted using dedicated image management software (D'z Image Viewer, CASIO, Japan).

The following trichoscopic parameters were assessed:

1. Hair diameter (HD)‐related parameters: maximum hair diameter (Max D), vellus hair count rate (VH%), indeterminate hair count rate (IH%), and terminal hair count rate (TH%).
2. Hair number per follicular unit (HN/FU)‐related parameters: single‐hair per follicular unit rate (1FU%), double‐hair per follicular unit rate (2FU%), three‐hair per follicular unit rate (3FU%), and multiple‐hair per follicular unit rate (MFU%).
3. Hair density‐related parameter: Total hair count within the 5 × 5 mm area (THC).

To elaborate further, HD was measured at the follicular base. To ensure accurate identification of individual hairs, visual tracing of each hair shaft was performed across the image, enabling reliable detection of overlapping, thin, and hypopigmented hairs. Manual counting was then conducted based on this tracing approach.

Hairs were classified as vellus (< 30 μm), indeterminate (30–60 μm), or terminal (≥ 60 μm). Hair follicles were considered to belong to the same follicular unit if the distance between them was ≤ 30 μm.

Image‐based measurements were conducted under blinded conditions. Quantitative analyses were performed using high‐resolution trichoscopic images by four experienced, professionally certified hair assessors. All measurements were independently performed by one of the four assessors, and a subset of cases was evaluated by all four assessors for inter‐assessor reliability analyses. To assess inter‐assessor reliability, the intraclass correlation coefficient (ICC) was calculated based on a subset of cases as described below.

### 2.5 Inter‐Assessor Reliability

Inter‐assessor reliability was evaluated using the ICC. This analysis was conducted retrospectively to evaluate measurement reliability. A subset of 15 patients was randomly selected, and measurements obtained at baseline and post‐treatment time points (total of 30 data points) were included. The ICC was calculated using a two‐way random‐effects model for absolute agreement [ICC (2, 1)].

### 2.6 Natural Course of MAGA

Using trichoscopic data from the untreated group, we analyzed the relationship between changes in visual appearance and changes in trichoscopic parameters in order to characterize the natural course of MAGA. The untreated group was categorized into H‐N C stages I–VII, and the measured values of each quantitative trichoscopic parameter were plotted as box plots to examine their distribution.

### 2.7 Quantitative Trichoscopic Evaluation System (QTES)

To reduce inter‐individual variability and to account for differences in parameter‐specific changes, we developed a scoring system that integrates selected trichoscopic values into a single composite score. We designate this scoring framework as the Quantitative Trichoscopic Evaluation System (QTES), and the composite score as the QTES Score.

To standardize individual parameter values, score categories were assigned based on the distribution of values observed across the untreated group patients and their correspondence with established H‐N C grades. Detailed scoring criteria are provided in Table 1.

**TABLE 1:** Predefined QTES scoring criteria.

Score	Max D	VH%	IH%	TH%	1FU%	3FU%	MFU%	THC
1	< 48.4	≦ 69.3	≦ 33.0	< 2.8	≦ 51.9	< 13.5	< 31.1	< 36.0
2	48.4–64.0	55.3–69.3	27.6–33.0	2.8–20.4	43.6–51.9	13.5–17.7	31.1–43.8	36.0–43.9
3	64.0–79.6	41.3–55.3	22.3–27.6	20.4–38.0	35.2–43.6	17.7–21.8	43.8–56.5	43.9–51.8
4	79.6–95.2	27.3–41.3	16.9–22.3	38.0–55.6	26.9–35.2	21.8–26.0	56.5–69.2	51.8–59.6
5	95.2–110.8	13.3–27.3	11.6–16.9	55.6–73.3	18.6–26.9	26.0–30.1	69.2–82.0	59.6–67.5
6	≦ 110.8	< 13.3	< 11.6	≦ 73.3	< 18.6	≦ 30.1	≦ 82.0	≦ 67.5

*Note:* Key quantitative trichoscopic parameters are highlighted in gray. “The value of A–B” means “greater than or equal to A, and less than B”. For example, “48.4–64.0” will be understood to mean “greater than or equal to 48.4 and less than 64.0”.

Abbreviations: 1FU%, single‐hair per follicular unit rate; 3FU%, triple‐hair per follicular unit rate; IH%, indeterminate hair count rate; Max D, maximum hair diameter; MFU%, multiple‐hair per follicular unit rate; QTES, quantitative trichoscopic evaluation system; TH%, terminal hair count rate; THC, total hair count within the 5 × 5 mm area; VH%, vellus hair count rate.

The scoring process was as follows:

1. Quantitative trichoscopic parameters (Max D, VH%, IH%, TH%, 1FU%, 2FU%, 3FU%, MFU%, THC) were measured and calculated using the untreated group dataset.
2. Parameters demonstrating a statistically significant correlation with visual appearance (Spearman's correlation coefficient *r*_*s*_ ≥ 0.4) were selected.
3. A correlation matrix was constructed to assess inter‐parameter correlations.
4. To standardize the measured values, each selected parameter was categorized into six score levels (1–6) based on its distribution.
  - The lowest 25% of values (seen in advanced MAGA, H–N C VII) were assigned a score of 1.
  - The highest 25% of values (seen in non‐severe MAGA, H–N C I) were assigned a score of 6.
  - The remaining values were evenly divided into four groups and assigned scores of 2–5.
5. Each patient's measured values were then converted into these 1–6 standardized scores.
6. The selected scores were summed to create the composite QTES Score.

The QTES framework was developed for the exploratory integration of multiple trichoscopic parameters, and its performance was subsequently examined in relation to clinical severity and treatment response.

### 2.8 Definition of Responders and Non‐Responders

Treatment response was defined based on changes in the QTES Score at baseline and 12 months. Patients demonstrating an increase or no change in the QTES Score were classified as responders, whereas those showing a decrease were classified as non‐responders. This cutoff was predefined based on prior evidence indicating that both HD and hair numbers naturally decline over time in untreated or placebo‐treated MGCM [18, 19]. Given the expected progression of MAGA, even a stable (unchanged) condition represents a clinically meaningful outcome. Accordingly, an unchanged QTES Score was interpreted as a favorable treatment response, reflecting prevention of the natural deterioration typically seen in MAGA.

### 2.9 Treatment Protocol of MGCM

Autologous MG were prepared from full‐thickness retro‐auricular occipital scalp tissue using mechanical disaggregation systems without enzymatic digestion. Detailed preparation procedures are provided in Supporting Information Methods S1.

Briefly, under local anesthesia with 1% lidocaine, three scalp tissue samples were obtained using a 3‐mm punch biopsy and processed according to the manufacturer's protocol to yield 6 mL of MG suspension for immediate use. No quantitative cellular characterization was performed in this clinical study. The suspension was injected into the subcutaneous layer at 0.1 mL per site with 1‐cm spacing within predefined treatment areas, reflecting the anatomical location of the anagen hair bulb in the subcutaneous layer. Injection sites were determined based on patient‐reported areas of concern and included the predefined vertex (hair‐whorl) evaluation region. Detailed preparation and injection procedures are described in Supporting Information Methods S1 and S2.

Of the 59 patients, 56 (94.9%) were treated using the Rigenera system and 3 (5.1%) using Medigraft; therefore, potential inter‐device variability was considered minimal.

Immediately following MG injection at the initial session, SHED‐CM was administered intradermally within the same treatment area to target the perifollicular region surrounding the follicular bulge (Supporting Information Methods S3). Additional SHED‐CM monotherapy sessions were performed at 3, 6, and 9 months after the initial combined treatment. Each session consisted of intradermal injections distributed evenly across the predefined treatment region. Further details of SHED‐CM preparation and administration are provided in Supporting Information Methods S4 and S5.

### 2.10 Safety Assessment

Adverse events were monitored throughout the study period and recorded at each visit.

### 2.11 Statistical Analysis

Prior to analysis, Max D, TH%, and MFU% were pre‐specified as key trichoscopic parameters for between‐group comparisons and treatment‐effect evaluation, based on their established relevance to HD and follicular unit composition.

Spearman's correlation analysis was performed to explore associations between H‐N C stages and trichoscopic values. For exploratory screening purposes, a threshold of *r*_*s*_ ≥ 0.4 was predefined to indicate moderate correlation. This cutoff was selected to identify potentially relevant parameters for further validation rather than to establish strong or definitive associations. A correlation matrix was created to identify multicollinearity. The Mann–Whitney U test was used to compare baseline characteristics between the untreated group and the MGCM group. The Wilcoxon signed‐rank test was used to compare before and after MGCM treatment. All tests were two‐tailed, with statistical significance set at *p* < 0.05.

## 3 Results

### 3.1 Baseline Characteristics

Baseline characteristics of the untreated group and the MGCM group are summarized in Table 2.

**TABLE 2:** Baseline characteristics of the study population.

	Untreated group (*N* = 133)		MGCM group (*N* = 59)	
Mean age (year‐old) ± SD	53.0 ± 11.0		53.9 ± 9.0	
Minimum‐maximum	21–86		34–86	
Hamilton‐Norwood classification	Number	% of group	Number	% of group
I	3	2.3	1	1.7
II	20	15.0	13	22.0
III	40	30.1	18	30.5
IV	26	19.5	13	22.0
V	20	15.0	10	16.9
VI	21	15.8	3	5.1
VII	3	2.3	1	1.7

*Note:* Percentages are calculated based on the total number of patients in each group.

Abbreviation: MGCM group, treatment group receiving micrografts and SHED‐CM.

### 3.2 Baseline Analysis

No statistically significant differences were detected between the untreated group and the MGCM group in pre‐specified key trichoscopic parameters including Max D, TH%, and MFU% (*p* = 0.347, 0.418, and 0.159, respectively). These findings indicate that the two groups were comparable at baseline with respect to major trichoscopic characteristics.

### 3.3 Inter‐Assessor Reliability

Inter‐assessor reliability was evaluated using the ICC. The ICC (2, 1) values ranged from 0.76 to 0.91 across parameters, with corresponding 95% confidence intervals indicating moderate to excellent inter‐assessor reliability. Detailed parameter‐specific results at pre‐ and post‐treatment are provided in Table S1.

### 3.4 Distribution of Individual Trichoscopic Parameters in Natural Course

**FIGURE 1:**
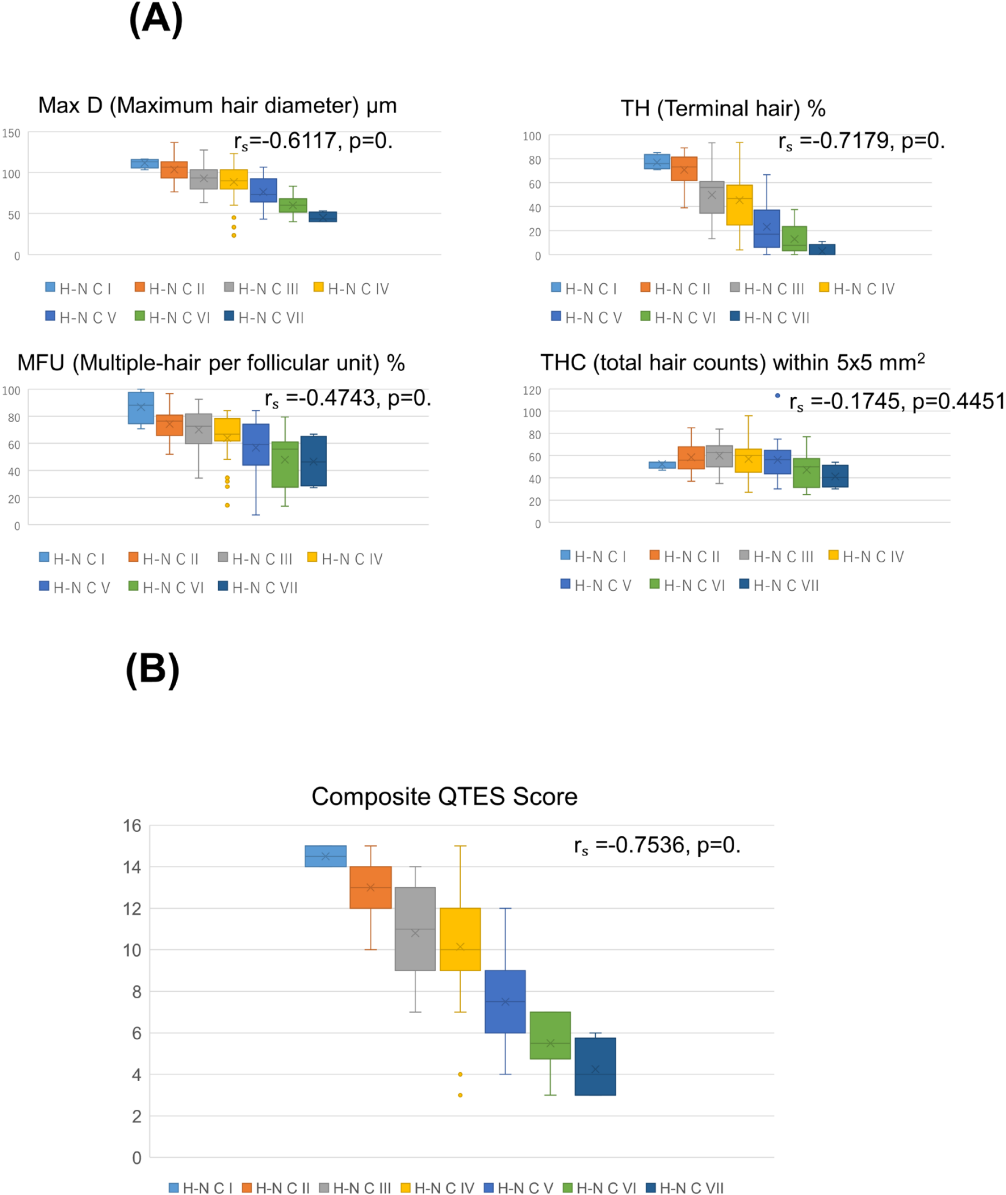
Distribution of trichoscopic parameters in the natural course (untreated group, *N* = 133) (A) Distribution of quantitative trichoscopic parameters (B) Distribution of composite QTES Score. H‐N C, Hamilton‐Norwood classification; QTES, quantitative trichoscopic evaluation system.

In the untreated group, multiple trichoscopic parameters were examined across H‐N C stages to characterize their behavior during the natural course of MAGA. As disease severity increased, several parameters related to HD and follicular unit composition showed consistent directional changes (Figure 1A). Specifically, parameters reflecting HD and HN/FU tended to decrease with advancing H‐N C stages, whereas those reflecting hair density remained relatively stable.

Spearman's rank correlation analysis (r_s_) further demonstrated that H‐N C stages strongly and negatively correlated with Max D, TH%, and MFU%, and positively correlated with VH% and 1FU% (Table S2). In contrast, 2FU% and THC did not show a significant correlation with clinical severity. Correlation matrix analysis showed strong inverse relationships between TH% and VH%, and between MFU% and 1FU% (Table S3). This indicates that, within each pair, one parameter largely mirrors the behavior of the other, meaning either can be used as a representative measure.

Accordingly, to minimize redundancy while retaining parameters most closely associated with visual appearance, Max D, TH%, and MFU% were selected as representative trichoscopic parameters for subsequent treatment effect analyses.

### 3.5 Distribution of Composite QTES Score in Natural Course

The distribution of the composite QTES Score (a composite score derived from the three representative parameters) demonstrated a stepwise decline with advancing H‐N C stages (Figure 1B), supporting its ability to reflect overall clinical severity by integrating multiple trichoscopic parameters. The composite QTES Score demonstrated a higher Spearman's correlation coefficient with H‐N C stages than the individual trichoscopic parameters. Detailed baseline composite QTES Scores and individual parameter scores according to H‐N C stage are presented in Table S4.

### 3.6 Individual‐Level Pre‐ and Post‐MGCM Treatment Comparison

**FIGURE 2:**
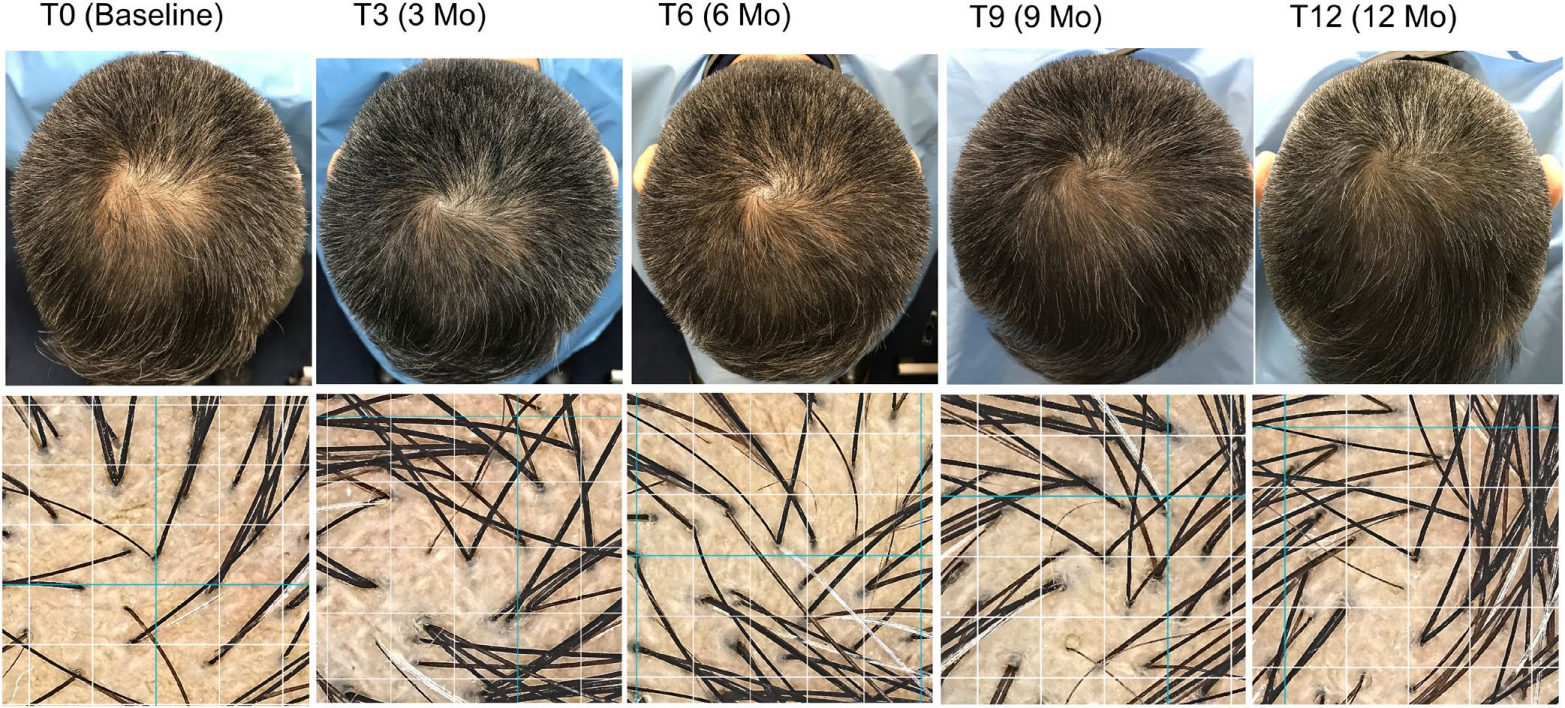
Longitudinal global and trichoscopic images across five time points. In the global images, the visible scalp area around the vertex appears to gradually decrease over time. In the trichoscopic images, there is a tendency for hairs to shift from single‐hair to multiple‐hair follicular units by T3 (3 months after MGCM), with MFUs appearing to be maintained thereafter. Trichoscopic images were obtained using polarized light to enhance follicular unit visibility, with a 1 × 1 mm grid overlaid. T0, baseline; T3, 3 months; T6, 6 months; T9, 9 months; T12, 12 months following MGCM treatment.

**FIGURE 3:**
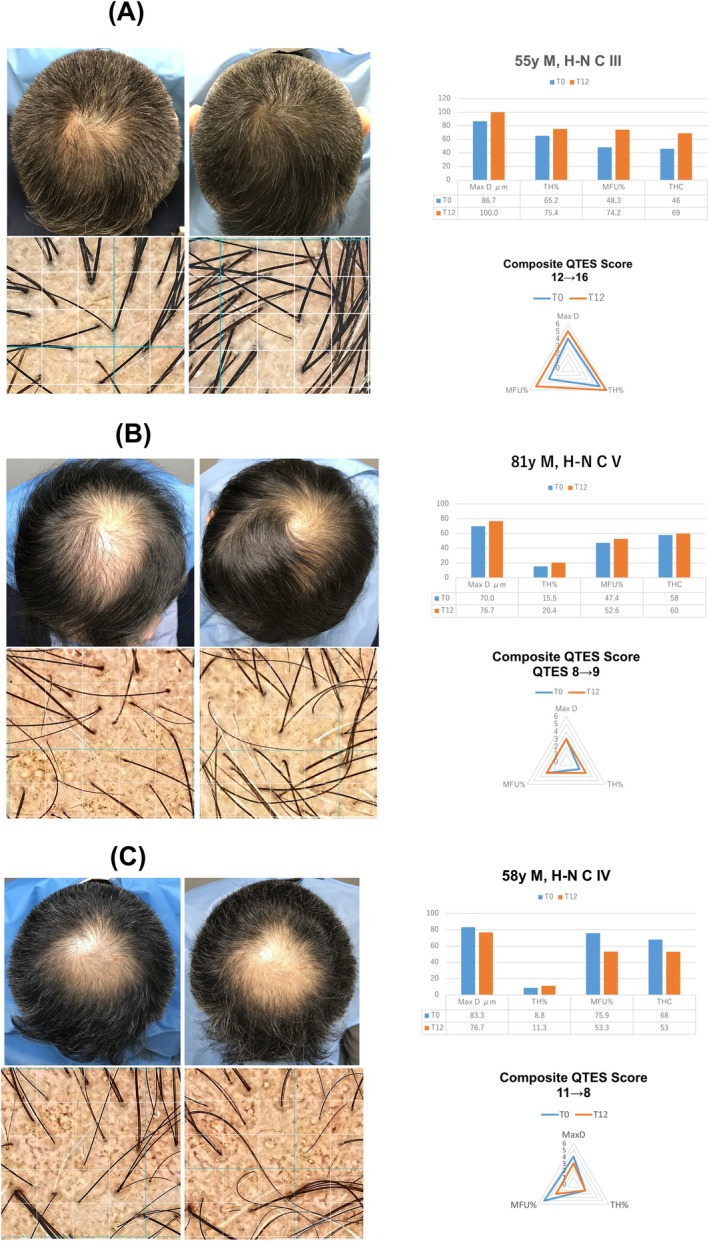
Individual‐level pre‐ and post‐MGCM treatment comparison. Representative cases (A–C) illustrate heterogeneous response patterns among patients, including responders and non‐responders. T0: baseline, T12: 12 months following treatment. Trichoscopic images were obtained using polarized light to enhance follicular unit visibility, with a 1 × 1 mm grid overlaid. (A) Responder, 55y, early stage; (B) Responder, 81y, advanced stage; (C) Non‐responder, 58y, advanced stage. H‐N C, Hamilton‐Norwood classification; Max, maximum hair diameter; MFU%, multi‐hair per follicular unit rate; QTES, quantitative trichoscopic evaluation system; TH%, terminal hair count rate; THC, total hair count within the 5 × 5 mm area.

Longitudinal global and trichoscopic images demonstrated progressive improvement in clinical appearance and trichoscopic findings over the 12‐month treatment period in representative responders (Figure 2). At the individual level, treatment responses to MGCM were heterogeneous. Some patients demonstrated concurrent improvement in visual appearance and multiple trichoscopic parameters (Figure 3A,B), whereas others showed no clear clinical improvement, with stable to slightly reduced appearance and declines in several parameters despite modest increases in selected measures (Figure 3C).

Radar chart visualization enabled simultaneous assessment of the magnitude and balance of score changes across parameters, illustrating whether improvements were global or parameter‐specific.

### 3.7 Group‐Level Pre‐ and Post‐MGCM Treatment Comparison

At the group‐level, the mean percentage changes in Max D, TH%, and THC showed an overall trend toward improvement. Max D increased significantly by 5.1% (*p* < 0.05), whereas TH% showed a greater but non‐significant increase of 10.2%. MFU% decreased slightly by 0.3%, and THC also exhibited a slight, non‐significant increase of 2.3%.

To further investigate the slight decrease in MFU%, a subgroup analysis was conducted comparing responders and non‐responders, as defined by changes in the composite QTES Score. This analysis demonstrated distinct response patterns between the two groups. In responders, Max D, TH%, and MFU% all showed significant increases (*p* < 0.05), whereas in non‐responders, MFU% showed a significant decrease (*p* < 0.05), with no significant changes observed in Max D or TH%. THC did not exhibit significant changes in either group. These findings suggest that the minimal overall change in MFU% at the group level reflects opposing directional changes between responders and non‐responders (Table 3).

**TABLE 3:** Group‐level pre‐ and post‐MGCM treatment comparison (*N* = 59).

	ALL		Responders		Non‐responders	
	T0	T12	T0	T12	T0	T12
Max D (μm)	88.0 ± 18.6	92.5 ± 17.5	86.4 ± 19.1	92.6 ± 15.2	93.2 ± 17.8	92.0 ± 21.7
% change	+5.1		+7.2		−1.3	
*p*‐value	0.00823*		0.00221*		0.60200	
TH% (%)	48.1 ± 26.9	53.0 ± 27.8	49.0 ± 26.8	56.5 ± 27.8	48.6 ± 27.0	45.5 ± 26.5
% change	+10.2		+15.3		−6.4	
*p*‐value	0.15154		0.02072*		0.24847	
MFU% (%)	68.3 ± 13.4	68.1 ± 13.2	65.7 ± 13.6	70.8 ± 11.8	74.4 ± 10.4	62.0 ± 13.9
% change	−0.3		+7.8		−16.7	
*p*‐value	0.33775		0.01652*		0.00214*	
THC (hairs per 5 × 5 mm^2^)	56.5 ± 12.4	57.8 ± 12.8	54.8 ± 13.1	58.1 ± 13.7	60.4 ± 9.6	58.2 ± 10.0
% change	+2.3		+6.0		−3.6	
*p*‐value	0.49152		0.05992		0.23393	

*Note:* Group‐level changes in quantitative trichoscopic parameters at baseline (T0) and 12 months after combined micrografts and SHED‐CM treatment (T12). Values are expressed as mean ± SD. Due to baseline values of zero in some cases, % change was calculated based on group means rather than individual values. Statistical comparisons were performed using Wilcoxon signed‐rank test.

Abbreviations: Max D, maximum hair diameter; MFU%, multiple‐hair per follicular unit rate; MGCM, combined treatment of micrografts and SHED‐CM; TH%, terminal hair count rate; THC, total hair count within the 5 × 5 mm area. Statistical comparisons were performed using Wilcoxon signed‐rank test: *p < 0.05.

### 3.8 Responder and Non‐Responder Analysis

Based on changes in the composite QTES Score, 69.5% of patients were classified as responders. Across H‐N C stages (II–VII), the proportion of responders was consistently higher than that of non‐responders (Table 4).

**TABLE 4:** MGCM response by Hamilton‐Norwood classification (*N* = 59).

	Responders		Non‐responders	
	Number	% of group	Number	% of group
	41	69.5	18	30.5
Hamilton‐Norwood classification		% within stage		% within stage
I	0	0	1	100
II	9	69.2	4	30.8
III	16	88.9	2	11.1
IV	7	53.8	6	46.2
V	6	60.0	4	40.0
VI	2	66.7	1	33.3
VII	1	100	0	0

*Note:* Percentages are calculated within MGCM group, or within each classification stage. Distribution of responders and non‐responders based on predefined QTES scoring criteria (Table 1) at 12 months after MGCM treatment. Responder status was defined according to changes in composite QTES Score.

Abbreviations: MGCM, combined treatment of micrografts and SHED‐CM; QTES, quantitative trichoscopic evaluation system.

**FIGURE 4:**
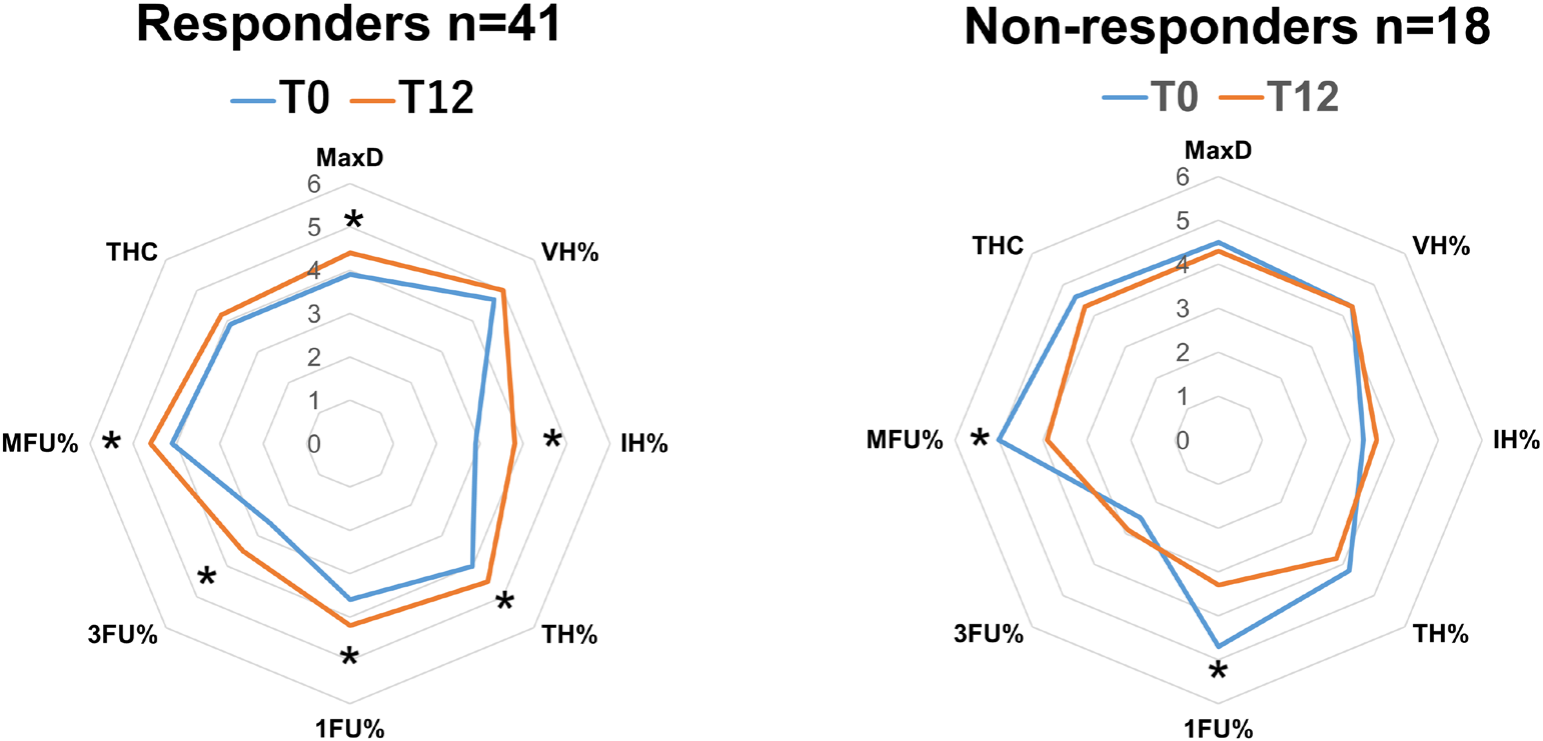
Group‐level pre‐ and post‐MGCM treatment score change (*N* = 59). Radar chart showing distinct patterns of trichoscopic parameter changes between responders and non‐responders, highlighting clear subgroup differences in multidimensional treatment response. Statistical analysis was performed using Wilcoxon signed‐rank test. 1FU%, single‐hair per follicular unit rate; 3FU%, triple‐hair per follicular unit rate; IH%, indeterminate hair count rate; Max D, maximum hair diameter; MFU%, multi‐hair per follicular unit rate; T0, baseline; T12, 12 months following treatment; TH%, terminal hair count rate; THC, total hair count within the 5 × 5 mm area; VH%, vellus hair count rate.

Within the responder group, pre‐ and post‐treatment comparisons revealed significant increases in Max D, IH%, TH%, 3FU%, and MFU%. In contrast, non‐responders showed a statistically significant worsening of 1FU% and MFU% (Figure 4).

Overall, responders showed improvements across both HD‐ and HN/FU‐related parameters, whereas non‐responders exhibited declines in key HN/FU‐related measures.

### 3.9 Safety

Treatment‐related adverse events were mild and self‐limited. Twenty patients (33.9%) experienced transient erythema, petechiae, or localized injection‐site pain; all symptoms resolved within three days. No serious adverse events were observed during the 12‐month follow‐up period.

## 4 Discussion

In this study, we evaluated the efficacy and safety of MGCM, a combination of autologous MG and allogenic SHED‐CM, and investigated which quantitative trichoscopic parameters best reflect visible changes in scalp appearance. MGCM was associated with clinically meaningful, appearance‐correlated trichoscopic changes in 69.5% of patients, without serious adverse events; although treatment responses were heterogeneous. Among the evaluated parameters, HN/FU emerged as a robust discriminator between responders and non‐responders, while the composite QTES Score showed the strongest overall association with visual scalp appearance. These results indicate that a multidimensional trichoscopic assessment provides a more reliable evaluation of treatment response than reliance on any single parameter. In addition, inter‐assessor reliability analysis demonstrated good to excellent agreement among evaluators, supporting the robustness of the quantitative trichoscopic measurements.

A notable finding was the limited relationship between hair density and visual scalp appearance. No significant pre‐to‐post treatment changes in hair density were observed in either responders or non‐responders, suggesting that hair density alone—despite its widespread use in prior clinical studies [1, 2, 4, 19, 20]—may not fully capture clinically meaningful or visually relevant changes. In contrast, parameters reflecting follicular unit composition were more informative, with HD and HN/FU showing closer alignment with visually assessed outcomes. This is consistent with a previous trichoscopic study demonstrating that parameters reflecting density heterogeneity (e.g., Max D, TH%, VH%) and follicular unit composition (e.g., 1FU%, MFU%) show stronger correlations with clinical severity than hair density alone [5, 21]. Furthermore, a previous study in patients with MAGA also demonstrated the efficacy of SHED‐CM monotherapy, as reflected by improvements in the composite score of HD and HN/FU [5].

**FIGURE 5:**
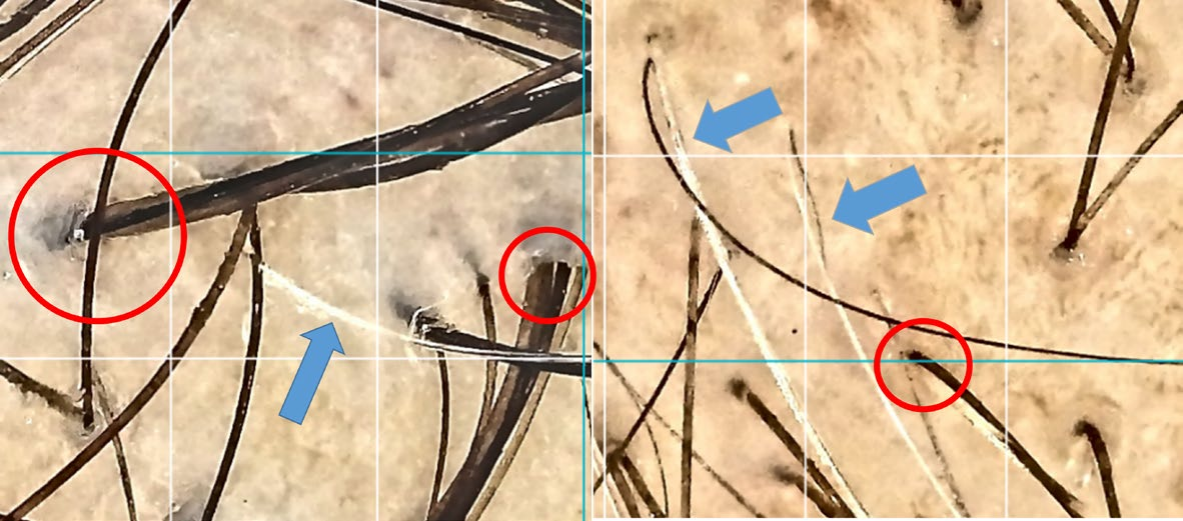
Representative examples of visual tracing for trichoscopic hair identification. Representative trichoscopic images illustrating the manual identification of individual hair shafts through visual tracing. This approach enables accurate detection of overlapping, thin, and hypopigmented hairs that may be difficult to distinguish using automated measurement methods. Red circles: Representative examples of overlapping hair shafts arising from a single follicular unit. Blue arrows: Hypopigmented (white) and thin hairs that may be difficult to accurately detect or distinguish using automated measurement approaches. Hair shafts were visually traced across magnified images to confirm continuity and ensure accurate inclusion in quantitative analysis.

The lack of significant change in hair density may partly reflect methodological differences, as fine or overlapping hairs may be more readily identified through manual assessment than by automated machine methods. In the present study, manual assessment incorporating visual tracing of individual hair shafts was used to identify overlapping, thin, and hypopigmented hairs. This approach may have allowed consistent inclusion of such hairs in the counts, even in areas affected by MAGA progression. As a result, changes in overall hair density may have been attenuated, whereas parameters reflecting follicular unit composition were more sensitive to treatment‐related changes. Representative examples of visual tracing are provided in Figure 5.

Several regenerative approaches for MAGA, including MG‐based approaches [1, 2, 3, 4], mesenchymal cell‐based therapies [9], fat grafts [20], SHED‐CM [5, 6], and platelet‐rich plasma (PRP) [22], have shown modest but measurable improvements in hair‐related parameters. Although direct comparisons are limited by heterogeneity in study design and outcome measures [1, 2, 4, 5, 9, 11, 20, 22], the magnitude of change observed in this study (Maximum HD: +5.1%, TH%: +10.2%) falls within the range reported in previous studies (HD: 2.3%–26.0%, TH%: 5.3%–26.3%). Notably, the present evaluation prioritized parameters more closely associated with visual scalp appearance [5, 21, 23] than hair density alone, which may partly explain differences compared with studies relying primarily on density‐based endpoints. These findings highlight the importance of selecting appropriate quantitative parameters when interpreting treatment effects.

Another key observation was the divergent behavior of HN/FU between responders and non‐responders. Responders demonstrated a significant increase in MFU%, whereas non‐responders showed a decline suggesting that HN/FU may serve as a sensitive indicator of treatment response. While the biological basis of this phenomenon cannot be directly established in the present study, increases in HN/FU may reflect partial reversal of follicular miniaturization [24, 25], improved follicular cycling dynamics [26, 27], or preservation of follicular unit integration [24, 25, 26, 27]. These interpretations remain speculative and could be addressed in future mechanistic investigations.

The observed increases in HD and HN/FU among responders are consistent with previously reported effects of the MG [2, 4, 9] and SHED‐CM [5] on the follicular microenvironment. In this context, although detailed cellular characterization was beyond the scope of this clinical study, the MG used in this study, derived from mechanically disaggregated full‐thickness scalp tissue, is expected to contain heterogeneous cellular components originating from epidermal, dermal, perifollicular, and subcutaneous compartments, along with extracellular matrix fragments that may contribute to regenerative signaling within the follicular microenvironment. In combination with bioactive factors present in SHED‐CM, these components may enhance follicular activity [6, 7, 8, 12] and microenvironmental conditions [7, 8, 10, 12]. In our dataset, increases in HD‐related parameters were evident, and responders also exhibited substantial improvement in HN/FU. This pattern aligns with earlier reports describing suppression of follicular miniaturization and structural recovery of the hair follicle [25, 26, 27]. Although the response rate in this study (69.5%) was comparable to that reported for SHED‐CM monotherapy [5] (75.8%), differences in study design and patient characteristics preclude direct comparison. The current study was not designed to assess superiority, but rather to explore potential complementary effects.

Conversely, the lack of response observed in some patients may reflect irreversible alterations of the follicular niche, as previously described in advanced MAGA [27]. However, the present study does not directly address mechanisms, and further studies may benefit from clarifying the biological pathways that may contribute to the multidimensional changes observed in this study.

To facilitate an integrated evaluation of multiple trichoscopic parameters, we introduced QTES, a composite scoring framework designed to reduce individual variability and account for differences in parameter scales. The composite QTES Score demonstrated a stronger association with visible scalp appearance than any single parameter and allowed stratification of patients into responders and non‐responders. QTES was developed as a pragmatic and exploratory tool based on correlations observed in the present dataset. Therefore, external validation in independent cohorts is required before broader application. It should also be noted that the predefined threshold of r_s_ ≥ 0.4 was intended for exploratory screening rather than for establishing strong associations. Accordingly, the identified relationships should be interpreted as hypothesis‐generating, and independent validation will be necessary to determine their robustness.

Representative clinical cases highlighted the heterogeneity of treatment responses and the clinical utility of a multidimensional assessment. The composite QTES Score provides an integrated evaluation of overall hair condition, while the individual parameter scores allow clinicians to assess the balance and relative changes between HD‐ and HN/FU‐related features. This framework may assist clinicians in deciding whether to continue, intensify, or modify treatment strategies, thereby supporting personalized management of hair‐regrowth therapy. Additionally, the size and shape of the triangular patterns displayed in the radar charts enable both clinicians and patients to identify which parameters improved or deteriorated, and to what extent.

While the present findings suggest potential therapeutic benefits, several limitations should be considered when interpreting the results.

First, the single‐center, non‐randomized single‐arm design without a placebo or control group limits causal inference and precludes separate evaluation of the individual contributions of MG and SHED‐CM. This study was therefore designed as an exploratory proof‐of‐concept to evaluate the combined therapeutic potential of MGCM. Second, a uniform washout period was not implemented, and residual carry‐over effects from prior therapies, including possible influences of short‐term treatment exposure, cannot be entirely excluded. Third, MG preparation was performed using two mechanical systems without quantitative characterization of cellular yield, which may introduce variability in cellular composition and biological activity. Fourth, evaluation was limited to predefined vertex regions and may not fully represent global scalp involvement. Fifth, individual follicle‐level tracking was not performed. Sixth, the sample size was relatively small (*N* = 59) and predominantly composed of mild‐to‐moderate cases, limiting the statistical power and restricting generalizability. Nevertheless, a limited number of patients at early (H‐N C I) and advanced (H‐N C VII) stages were intentionally included to enable preliminary observations across a broader spectrum of disease severity, rather than confining the analysis to intermediate stages alone. Finally, inter‐assessor reliability analysis was conducted retrospectively without prespecification in the study protocol. In addition, fully blinded external assessment by independent evaluators was not implemented, which may introduce potential bias.

Our findings suggest that the combination of MG and SHED‐CM is a promising approach for MAGA. Given their potentially complementary mechanisms, future strategies may benefit from patient‐specific approaches based on vascular remodeling capacity, residual follicular stem cell reserve, and disease severity.

To address the limitations of the present study, future investigations should employ randomized controlled designs with appropriate comparator groups to enable causal inference and to clarify the individual contributions of MG and SHED‐CM. Standardization of treatment protocols, including predefined washout conditions and quantitative characterization of MG preparations, will be important to minimize potential carry‐over effects, reduce variability, and improve reproducibility.

In addition, larger, multicenter studies with broader disease representation, together with blinded outcome assessments, are warranted to enhance generalizability and minimize bias. Incorporation of multi‐regional scalp evaluation and, where feasible, follicle‐level longitudinal analysis may further strengthen the robustness and clinical relevance of future findings.

In summary, MGCM appears to be a well‐tolerated modality associated with clinically meaningful improvement in a substantial proportion of patients with MAGA. Changes in HN/FU together with the composite QTES Score provide a more comprehensive evaluation of treatment response than single‐parameter approaches, supporting the use of integrated quantitative trichoscopic analysis—including HD and HN/FU—in both clinical practice and research.

## Author Contributions

T.K. designed the study, collected and analyzed the data, and wrote the manuscript. A.M. contributed to data collection and analysis. C.H. contributed to data analysis. J.T. encouraged T.K. to investigate, and supervised the project.

## Funding

The authors have nothing to report.

## Ethics Statement

This study was approved by Tokyo Midtown Medical Center Ethics Review Board (倫‐2021‐03/May 21, 2021) and registered with the University Hospital Medical Information Network Clinical Trial Registry (UMIN000045897/October 28, 2021).

## Consent

All subjects agreed to participate in this study, use for publication of images, and provided written informed consent.

## Conflicts of Interest

The authors declare no conflicts of interest.

## Supporting information


**Data S1:** jocd70982‐sup‐0001‐Supinfo.docx.

**Table S1:** Inter‐assessor reliability of trichoscopic parameters.

**Table S2:** Correlations between clinical stages and trichoscopic parameters in untreated group (*N* = 133).

**Table S3:** Correlation matrix of quantitative trichoscopic values in untreated group (*N* = 133).

**Table S4:** QTES baseline scores in untreated group (*N* = 133).

## Data Availability

The data that support the findings of this study are available on request from the corresponding author.
