# Supplementary material for: Hair Number per Follicular Unit as a Marker of Treatment Response to Combined Autologous Scalp‐Derived Micrografts and Allogeneic SHED‐CM in Male Androgenetic Alopecia
Source: J Cosmet Dermatol. 2026 Jun 17;25(6):e70982. doi: 10.1111/jocd.70982 (PMC13276026; doi:10.1111/jocd.70982)
Supplement: Supplementary file 1 — Data S1: jocd70982‐sup‐0001‐Supinfo.docx. [file JOCD-25-e70982-s003.docx]

The following detailed procedures are provided to ensure reproducibility of the minimally manipulated autologous scalp-derived MG preparation.

**Supplementary Methods S1.**

**Donor Tissue Harvesting**

Under local anesthesia with 1% lidocaine, three full-thickness scalp tissue specimens were harvested from the retro-auricular occipital region using a sterile 3-mm punch biopsy instrument. Each specimen included epidermis, dermis, and subcutaneous tissue.

The harvested tissue samples were immediately processed under sterile conditions. No enzymatic digestion or ex vivo cell expansion procedures were performed.

**Supplementary Methods S2.**

**Mechanical Disaggregation and Suspension Preparation**

MG were prepared using either the Rigenera system (Human Brain Wave, Turin, Italy) or the Medigraft system (Medigraft FZCO, Dubai, UAE), according to the respective manufacturer’s instructions.

Each biopsy specimen was subdivided into smaller fragments prior to mechanical disaggregation. Fragmentation was performed in sterile saline solution (2.0 mL per processing cycle). Multiple sequential disaggregation cycles were conducted to maximize tissue processing efficiency.

The resulting suspension was centrifuged at manufacturer-specified low gravitational force conditions to separate larger debris from the injectable supernatant fraction. The final collected supernatant constituted the MG suspension, with a total injectable volume of approximately 6 mL per patient.

The procedure was designed to have minimal mechanical manipulation. No quantitative cellular characterization was performed in this clinical study.

**Supplementary Methods S3.**

**MG Injection Technique**

Following local anesthesia of the recipient area with 1% lidocaine, the MG suspension was injected into the subcutaneous layer using a 30-gauge needle attached to a 1-mL Luer-Lok® (Becton Dickinson, Ontario, Canada).

Injections were administered at 0.1 mL per site with approximately 1-cm spacing, corresponding to roughly 60 injection points and a theoretical coverage area of approximately 60 cm² per session.

Injection sites were confined to clinically active thinning regions and included the predefined vertex (hair-whorl) evaluation site. Outcome measurements were subsequently performed within these predefined treatment areas to ensure anatomical consistency between intervention and evaluation.

**Supplementary Methods S4.**

**SHED-CM Preparation**

The SHED-CM product (SGF; Solaria, Tokyo, Japan) used in this study is a standardized commercial preparation. SHED were isolated from exfoliated deciduous teeth obtained from healthy donors aged 6–12 years who underwent rigorous medical screening, including testing for HIV, HBV, HCV, HTLV-1/2, parvovirus B19, and syphilis.

Cells were cultured in Dulbecco’s Modified Eagle’s Medium (DMEM) supplemented with 10% fetal calf serum under controlled conditions and expanded up to passage 4 prior to conditioned medium collection.

Phenotypic characterization was performed by flow cytometry analysis. SHED expressed mesenchymal stem cell markers (CD90, CD73, CD105) and lacked expression of hematopoietic/endothelial markers (CD45, CD34, CD11b, HLA-DR). In addition, the cells demonstrated adipogenic, chondrogenic, and osteogenic differentiation capacity, consistent with the minimal criteria for mesenchymal stem cells defined by the International Society for Cellular Therapy.

Manufacturing was conducted in a certified Cell Processing Center (CPC) under Good Manufacturing Practice (GMP)-grade conditions for clinical application. As part of quality control procedures, endotoxin testing and bacterial contamination testing were routinely performed prior to product release.

The concentration of CD63-positive extracellular vesicles (50–150 nm), corresponding to exosome-sized vesicles, was quantified at 2.3 × 10⁹ particles/mL. The final SHED-CM product was cell-free and underwent sterilization and quality control testing before clinical use.

**Supplementary Methods S5.**

**SHED-CM Administration and Treatment Schedule**

Immediately after MG injection during the initial treatment session, SHED-CM was administered in the upper dermis within the same treatment area using a 32-gauge turtle-pin needle (JM Biotech, Seoul, Korea).

Each SHED-CM session consisted of a total volume of 4.5 mL evenly distributed across the affected scalp region.

Additional SHED-CM monotherapy sessions were performed at 3, 6, and 9 months following the initial MGCM treatment. Clinical outcomes were evaluated 3 months after the final session (i.e., 12 months after the initial combined treatment).
