# Supplementary material for: Hair Number per Follicular Unit as a Marker of Treatment Response to Combined Autologous Scalp‐Derived Micrografts and Allogeneic SHED‐CM in Male Androgenetic Alopecia
Source: J Cosmet Dermatol. 2026 Jun 17;25(6):e70982. doi: 10.1111/jocd.70982 (PMC13276026; doi:10.1111/jocd.70982)
Supplement: Supplementary file 2 — Table S1: Inter‐assessor reliability of trichoscopic parameters. [file JOCD-25-e70982-s001.pdf]

Supplementary TABLE 1

Inter-assessor reliability of trichoscopic parameters

	T0	Interpretation	T12	Interpretation
Max D	0.907	excellent	0.850	good
TH%	0.881	good	0.913	excellent
MFU%	0.840	good	0.760	good
THC	0.878	good	0.910	excellent
QTES	0.901	excellent	0.879	good

ICC (2,1) was calculated based on a randomly selected subset of 15 patients (30 data points) evaluated by four independent assessors. T0 indicates baseline, and T12 indicates 12 months after MGCM (micrografts and SHED-CM) treatment.

Abbreviations: Max D, maximum hair diameter; MFU%, multiple-hair per follicular unit rate; TH%, terminal hair count rate; THC, total hair count within the 5x5 mm area.
