# Supplementary material for: Hair Number per Follicular Unit as a Marker of Treatment Response to Combined Autologous Scalp‐Derived Micrografts and Allogeneic SHED‐CM in Male Androgenetic Alopecia
Source: J Cosmet Dermatol. 2026 Jun 17;25(6):e70982. doi: 10.1111/jocd.70982 (PMC13276026; doi:10.1111/jocd.70982)
Supplement: Supplementary file 3 — Table S2: Correlations between clinical stages and trichoscopic parameters in untreated group (N = 133). [file JOCD-25-e70982-s004.pdf]

Supplementary TABLE 2

Correlations between clinical stages and trichoscopic parameters in untreated group (N=133)

Parameter		r_s	p-value	$r_s>0.6$	$0.4<r_s<0.6$	$0.1<r_s<0.4$	NS
HD-related	Max D	-0.61117	0.	◎			
	VH%	0.65616	0.	◎			
	IH%	0.37962	1.00E-05			△	
	TH%	-0.71791	0.	◎			
HN/FU-related	1FU%	0.46876	0.		○		
	2FU%	-0.11375	0.19235				×
	3FU%	-0.39327	0.			△	
	MFU%	-0.47426	0.		○		
Hair density-related	THC	-0.17453	0.44510				×

HD-related parameters and HN/FU-related parameters showed strong correlations with clinical stage, whereas hair density-related parameters did not.

Abbreviations: 1FU%, single-hair per follicular unit rate; 2FU%, double-hair per follicular unit rate; 3FU%, triple-hair per follicular unit rate; HD-, hair diameter; HN/FU-, hair number per follicular unit; IH%, indeterminate hair count rate; Max D, maximum hair diameter; MFU%, multiple-hair per follicular unit rate; TH%, terminal hair count rate; THC, total hair count within the 5x5 mm area; VH%, vellus hair count rate.
