# Supplementary material for: Hair Number per Follicular Unit as a Marker of Treatment Response to Combined Autologous Scalp‐Derived Micrografts and Allogeneic SHED‐CM in Male Androgenetic Alopecia
Source: J Cosmet Dermatol. 2026 Jun 17;25(6):e70982. doi: 10.1111/jocd.70982 (PMC13276026; doi:10.1111/jocd.70982)
Supplement: Supplementary file 4 — Table S3: Correlation matrix of quantitative trichoscopic values in untreated group (N = 133). [file JOCD-25-e70982-s005.pdf]

Supplementary TABLE 3

Correlation matrix of quantitative trichoscopic values in untreated group (N=133)

	Max D	VH%	IH%	TH%	1FU%	2FU%	3FU%	MFU%	THC
Max D	1								
VH%	-0.70181	1							
IH%	-0.32218	0.04302	1						
TH%	0.74135	-0.81887	-0.60519	1					
1FU%	-0.39946	0.37581	0.05439	-0.32238	1				
2FU%	0.03968	-0.16317	0.04761	0.09175	-0.39051	1			
3FU%	0.35671	-0.23975	-0.13915	0.26639	-0.58072	-0.27503	1		
MFU%	0.38788	-0.39869	-0.06397	0.34633	-0.93085	0.37530	0.57677	1	
THC	0.21900	-0.03909	0.10888	-0.02847	-0.53826	0.05811	0.45730	0.51668	1

Cells with an absolute correlation coefficient of 0.8 or higher are shaded in gray. VH% and TH% were highly correlated, as were 1FU% and MFU%. These findings suggest that VH% and TH% are interchangeable in assessing HD, while both 1FU% and MFU% are equally relevant for evaluating HN/FU**.**

Abbreviations: 1FU%, single-hair per follicular unit rate; 2FU%, double-hair per follicular unit rate; 3FU%, triple-hair per follicular unit rate; IH%, indeterminate hair count rate; Max D, maximum hair diameter; MFU%, multiple-hair per follicular unit rate; TH%, terminal hair count rate; THC, total hair count within the 5x5 mm area; VH%, vellus hair count rate.
