# Supplementary material for: Hair Number per Follicular Unit as a Marker of Treatment Response to Combined Autologous Scalp‐Derived Micrografts and Allogeneic SHED‐CM in Male Androgenetic Alopecia
Source: J Cosmet Dermatol. 2026 Jun 17;25(6):e70982. doi: 10.1111/jocd.70982 (PMC13276026; doi:10.1111/jocd.70982)
Supplement: Supplementary file 5 — Table S4: QTES baseline scores in untreated group (N = 133). [file JOCD-25-e70982-s002.docx]

Supplementary TABLE 4

QTES baseline scores in untreated group (N=133)

| H-N C | Max D | TH% | MFU% | QTES Score |
| --- | --- | --- | --- | --- |
| I | 5.8 | 5.8 | 5.3 | 16.9 |
| II | 5.0 | 5.4 | 4.0 | 14.4 |
| III | 4.3 | 4.2 | 3.5 | 12.0 |
| IV | 4.1 | 3.8 | 3.0 | 10.9 |
| V | 3.3 | 2.6 | 2.4 | 8.3 |
| VI | 2.3 | 2.1 | 1.5 | 5.9 |
| VII | 1.3 | 1.3 | 1.3 | 3.9 |

**Measured parameters**: Max D: Maximum hair diameter, TH%: Terminal hair count rate, MFU%: Multiple-hair per follicular unit rate, THC: Total hair count within and on the 5x5 mm grid, QTES Score: Average total score of three representative trichoscopic factors, QTES: Quantitative Trichoscopic Evaluation System,

QTES scores were calculated based on the parameters presented in Table 1.

**Note**: A trend toward higher QTES scores in early stages and lower scores in advanced stages was observed.
